# Heterostructured ZnCdS@ZIF-67 as a Photocatalyst for Fluorescent Dye Degradation and Selectively Nonenzymatic Sensing of Dopamine

**DOI:** 10.3390/ma15217683

**Published:** 2022-11-01

**Authors:** Zhichao Wang, Bianying Wen, Jie Zhou, Xin Zhao, Xiaoyuan Zhang, Zhiqiang Su

**Affiliations:** 1 Precision Forestry Key Laboratory of Beijing, Beijing Forestry University, Beijing 100083, China; 2 Key Laboratory of Processing and Quality Evaluation Technology of Green Plastics of China National Light Industry Council, Beijing Technology and Business University, Beijing 100048, China; 3 State Key Laboratory of Chemical Resource Engineering, Beijing Key Laboratory of Advanced Functional Polymer Composites, Beijing University of Chemical Technology, Beijing 100029, China

**Keywords:** photocatalytic degradation, ZnCdS, ZIF-67, Rhodamine B, dopamine sensor

## Abstract

Dopamine (DA) plays the role of the transmitter of information in the brain. Neurological diseases and depression are in close relationship with DA release. In this study, we developed a co-catalyst Zn_0.2_Cd_0.8_S@zeolitic imidazolate framework-67 (Zn_0.2_Cd_0.8_S@ZIF-67) to improve the photocatalyst efficacy of Rhodamine B (RhB) and electrochemical sensing of DA. Results show that Zn_0.2_Cd_0.8_S@ZIF-67 exhibits optimal photocatalytic activity with the addition of 80 mg ZIF-67. The degradation percentage of RhB by Zn_0.2_Cd_0.8_S@ZIF-67 reached 98.40% when the co-catalyst was 50 mg. Radical trapping experiments show that ·O_2_^−^ played a significant role in the photocatalytic degradation of RhB. The catalytic mechanism of the Zn_0.2_Cd_0.8_S@ZIF-67 was found as a Z-type photocatalysis. Finally, a DA biosensor was constructed and displayed a high response and selectivity to DA. This can be attributed to the heterojunction between Zn_0.2_Cd_0.8_S and ZIF-67, which can significantly enhance the separation of e^−^/h^+^ and improve charge transfer. These findings will play a positive role in the in-situ monitoring of neurological diseases and depression.

## 1. Introduction

Dopamine (DA) is the most abundant catecholamine neurotransmitter in the brain. It plays an important role in the central nervous system, kidneys, cardiovascular system, and endocrine system [1]. DA levels can affect heart rate, leading to heart failure and Parkinson’s disease [2]. Therefore, in situ measurement of DA content can be obtained using biosensors. Among them, electrochemical monitoring of DA is an excellent strategy [3]. Lee et al. [2] designed a ruthenium nanoparticle-immobilized multiscale pore-containing carbon nanofiber-based field-effect transistor nonenzymatic sensor to detect DA. They found that the porous carbon structure not only promoted the generation of small-size Ru particles, but also induced a large active surface area for DA. Dashtian et al. [4] formed heterojunction by coupling dendrite-like CuInSe_2_(CIS) with Cu_2_O nanoparticles. This PEC sensing system targeted DA within two working ranges from 0.1–5.5 μM and 5.5–150 μM at a low detection limit of 2.1 nM. Cu_2_O/CIS acted as photoelectric convertors and amplifier layers for PEC-sensing. The signal quenching was obtained in the presence of DA due to a hindrance effect.

Metal ions and organic ligands can form metal-organic framework (MOF) materials with unique porous structures and large surface areas [5]. Due to the diversity of MOFs, the photoadsorption properties can be controlled by constructing organic ligands and metal ions [6,7,8]. Most metal nodes, such as Zr-O, Ti-O, and Cu-O clusters, do not have a visible light response. Therefore, metals responsive to visible light can be substituted or doped to provide MOF with an excellent light trapping ability in the visible region. On the one hand, the energy band structure of MOFs needs to be fine-tuned to meet the reduction potential of the reaction. On the other hand, most MOFs are microporous, which allows the selectivity and limited reactivity to be regulated by the diffusion of the catalyst. Multi-stage porous MOFs can reduce the mass transfer resistance and hopefully allow for the conversion of larger reactants. Ngoc Tran et al. [9] fabricated gold nanoparticles/metal–organic frameworks (Au NPs/IRMOF-3) hybrids. Au NPs/IRMOF-3 multilayers generated chemical and electromagnetic amplification of Raman signals of RhB at a low concentration and high enhancement factor. This hybrid can be used for DA sensing with high sensitivity and stability (limit of detection is 1.42 × 10^−18^ M with a wide linear range from 10^−17^ to 6 × 10^−8^ M of DA).

Developing efficient photocatalytic systems depends mainly on designing and fabricating appropriate material combinations. They are usually combined with other semiconductors and metallic materials to reduce the agglomeration of different materials for efficient electron transfer [10]. Zhang et al. [11] prepared the Ag/UiO-66-NH_2_ by soaking AgNO_3_ into zirconium 1,4-dicarboxybenzene MOF (UiO-66) and then reducing it by NaBH_4_. The photodegradation of Rhodamine B (RhB) dye by Ag/UiO-66-NH_2_ under ultraviolet and visible light was better than that of the original UiO-66-NH_2_. The built-in heterostructure accelerates electron-hole separation and absorption of visible light. Zeolitic imidazolate frameworks (ZIFs) molecular sieve comprises Zn^2+^, Co^2+^, and imidazole ligands. ZIF-67 is composed of metal ions Co^2+^ and 2-Methylimidazole. The broad surface area of ZIF-67 exposes more active sites in the photocatalysis process. Dong et al. [12] designed a WO_3_/CsPbBr_3_/ZIF-67 ternary heterostructure for efficient CO_2_ photoreduction. The surface modification of zeolites with active Co sites in ZIF-67 promoted the adsorption and activation of CO_2_. The synergistic effect of charge separation and CO_2_ absorption greatly enhanced the photocatalytic activity. Kumar et al. [13] prepared a graphite nitride Ag@ZIF-67 complex and investigated its degradation of Methylene blue and Congo red (CR) dyes.

CdS has been regarded as a unique photocatalyst among II–VI compound semiconductors. The photodegradation efficacy of CdS is based on the dopant elements [14], such as Fe, Co, and Ni. Cd-S linkage, along with other functional groups in the spherical nanoparticles of Fe-CdS, was demonstrated [15]. The Fe-doped CdS NPs showed superior catalytic potential compared to undoped CdS. Zn_x_Cd_1−x_S is a ternary photocatalyst with a controllable band gap and band edge position. It is necessary to find strategies to compensate for the deficiencies of ZnCdS and produce a better photocatalytic performance. Nanomaterials are combined with ZnCdS and reduce the occurrence of electron-hole pair recombination to a certain extent. Zhang et al. [16] synthesized MOF-derived porous hollow carbon nanobubbles@ZnCdS multi-shelled dodecahedral cages (C@ZnCdS MSDCs). C@ZnCdS MSDCs displayed superior photoelectrochemical (PEC) performance. The photoexcited electrons were transferred from the conduction band (CB) of C@ZnCdS MSDCs to the CB of TiO_2_. Simultaneously, holes were transferred from the valence band (VB) of TiO_2_ to the VB of C@ZnCdS MSDCs, leading to a boost of visible-light response and separation of photogenerated e^−1^/h^+^. 

**Scheme 1:**
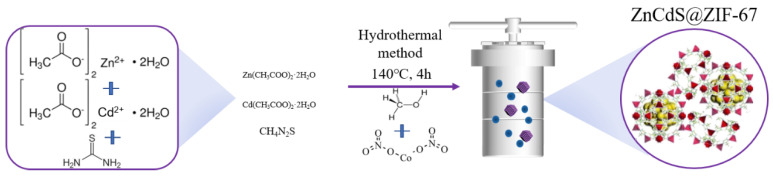
Schematical drawing of Zn_0.2_Cd_0.8_S@ZIF-67 via one-pot hydrothermal method.

In this study, we designed a Zn_0.2_Cd_0.8_S@ZIF-67 co-catalyst to form a heterojunction via a one-pot hydrothermal method (Scheme 1). Zn_0.2_Cd_0.8_S has excellent photocatalytic performance [17,18,19,20]. ZIF-67 promoted the exposure of active sites in catalytic reactions and the effect of Co on electrical conductivity. The combination of ZnCdS and ZIF-67 is supposed to play a synergistic effect in strengthening photocatalytic performance. In addition, the photocatalytic activity of Zn_0.2_Cd_0.8_S@ZIF-67 was evaluated by degrading the RhB solution and functioning as a DA sensor. Free radical trapping experiments were carried out to investigate the active substances, which play a significant role in the determination of selective sensing of DA.

## 2. Materials and Methods

### 2.1. Materials

The materials and reagents are as follows: 2-Methylimidazole (99%, J&K, Beijing, China), thiourea (99%, J&K, Beijing, China), potassium bromide (99%, J&K, Beijing, China), zinc dihydrate acetate (99%, J&K, Beijing, China), sodium sulfate (99%, J&K, Beijing, China), disodium ethylenediaminetetraacetic acid (EDTA) (99%, J&K, Beijing, China), benzoquinone (99%, J&K, Beijing, China), methanol (≥99.7%, J&K, Beijing, China), isopropanol (99.5%, J&K, Beijing, China), Rhodamine B (96%, J&K, Beijing, China), anhydrous ethanol (≥99.7%, Fuyu chemical, Tianjin, China), cobalt nitrate hexahydrate (98%, Damao chemical reagent factory, Tianjin, China), and cadmium acetate dihydrate (99%, Thermo Fisher Scientific, Waltham, MA, USA). The ITO slides are purchased from Luoyang Guluo Glas Co., Ltd. (Luoyang, China).

### 2.2. Synthesis of ZIF-67

Solution A was prepared by dissolving 1.45 g cobalt nitrate hexahydrate in 50 mL of methanol by agitation. We dissolved 3.2 g 2-Methylimidazole in 50 mL of methanol to form solution B. Solutions A and B were stirred for 24 h, then centrifuged with a low-speed centrifuge, and washed with ethanol and ultra-pure water. The purple precipitates were dried for 12 h in an oven at 60 °C. The product was ground into powder for subsequent use.

### 2.3. Synthesis of Zn_0.2_Cd_0.8_S@ZIF-67

Zn_0.2_Cd_0.8_S@ZIF-67 was prepared using the one-pot method. Specifically, 0.22 g of zinc dihydrate, 1.07 g of cadmium dihydrate, 0.38 g of thiourea, and different amounts (10, 30, 50, 70 mg) of ZIF-67 were dissolved in 80 mL of water. The mixed solution was ultrasonically treated for about 30 min and magnetically stirred for 30 min. The solution was then transferred to the lining of the Teflon autoclave, and treated at 140 °C for about 4 h. After the reaction was completed, the reactor was slowly cooled to room temperature. The product was then centrifuged, washed with water and ethanol, and dried for 12 h in an oven at 60 °C. The product was ground into powder for subsequent use.

### 2.4. Material Characterization

Information about the microscopic topography of the photocatalyst was obtained from the scanning electron microscope (SEM) images by using a Hitachi SU8010 SEM. The microstructure was characterized by the FEI-TALOS-F200X transmission electron microscope (TEM). From the Fourier transform infrared spectrometer (FTIR-650), information about the functional groups carried by the catalyst were obtained. The test wavelength range is set from 4000 to 400 cm^−1^. An Ultima IV X-ray diffractometer (XRD) was used to analyze the structure of the samples. The element properties were studied by Thermo Scientific K-alpha+X-ray photoelectron spectroscopy (XPS). PerkinElmer and LAMADA950 ultraviolet–visible spectroscopy (UV-vis) provided UV absorption spectra and UV diffuse reflectance spectra. The ultraviolet diffuse reflectance spectroscopy (UV-vis DRS) displayed information about the light absorption by photocatalysts. Steady-state, transient fluorescence spectra of samples were obtained with a Hitachi F-4600 photometer. The Mott–Schottky curve, photocurrent response, and Electrochemical Impedance Spectroscopy (EIS) Nyquist plot were measured on an electrochemical workstation (CHI760E).

### 2.5. Photocatalytic Degradation Experiment

The experimental procedure was as follows: 5 mg of RhB powder was dissolved it in 500 mL ultra-pure water, 10 mg/L of RhB solution was obtained, and each photocatalytic reactor was filled with 50 mL of RhB solution. A total of 30 mg of photocatalyst was dispersed in the RhB solution with a magnetic stirrer in a 100 mL beaker. The catalyst sample was stirred at a constant speed for half an hour without light to reach an equilibrium point between the dye and the catalyst. With a 500 W Xe lamp, the 50 mL of dye solution was centrifuged every 30 min. After centrifugation, the RhB solution was tested for instant absorbance. The photocatalytic degradation efficiency (C/C_0_) of RhB required its absorbance to be converted. 

### 2.6. Free Radical Trapping Experiment

The RhB degradation by catalyst was characterized by a free radical trapping experiment. Ethylenediaminetetraacetic acid disodium (EDTA-2Na) was used as a trapping agent for h^+^, while benzoquinone (BQ) was used for benzoquinone (·O^2−^) and isopropanol (IPA) was used for hydroxyl radicals (·OH). A total of 30 mg of photocatalyst was dispersed in 50 mL of RhB solution with a 10 mg/L concentration. Then, the 50 mL of trapping agent was added to the degradation container, and the samples were stirred at a constant speed for half an hour in the dark to mix RhB and the catalyst before the actual illumination. 

### 2.7. Fabrication of the DA Sensor

The fabrication of the photoelectrochemical (PEC) sensor was as follows: Indium Tin Oxide (ITO) electrodes were sonicated in acetone, absolute ethanol, and double distilled water for 10 min and then dried at room temperature. The 6 mg of Zn_0.2_Cd_0.8_S@ZIF-67 was then dispersed in a mixed solution of 45 μL ethanol and 5 μL 0.1% (*w*/*w*) Nafion, which was sonicated for 20 min. Then, 15 μL suspension droplets were drop-casted on the 1 cm×1 cm square centimeter ITO surface and dried at room temperature to obtain the ITO/Zn_0.2_Cd_0.8_S@ZIF-67 electrode. The modified ITO electrode was used as the working electrode, the platinum plate electrode was used as the paired electrode, and the Ag/AgCl (saturated KCl solution) was used as a reference electrode. With the 500 W Xe lamp, the photoelectrochemical responses of the modified electrode to DA in 0.1 M phosphate buffered saline (PBS) solution were evaluated by chronoamperometry.

For the DA selectivity test, we selected Fructose, Lactic acid (LA), ascorbic acid (AA), sulfuric acid (UA), sucrose, glucose, and lactose as the interfering molecules for amperometric detection of DA using the CHI760E platform. These molecules co-exist in human blood plasma. The concentration of DA was 100 μM in 0.1 M PBS. The additional amount of interfering molecules was 100 μM in 0.1 M PBS. 

## 3. Results and Discussion

### 3.1. Morphological and Structural Characterizations of Zn_0.2_Cd_0.8_S@ZIF-67

**Figure 1:**
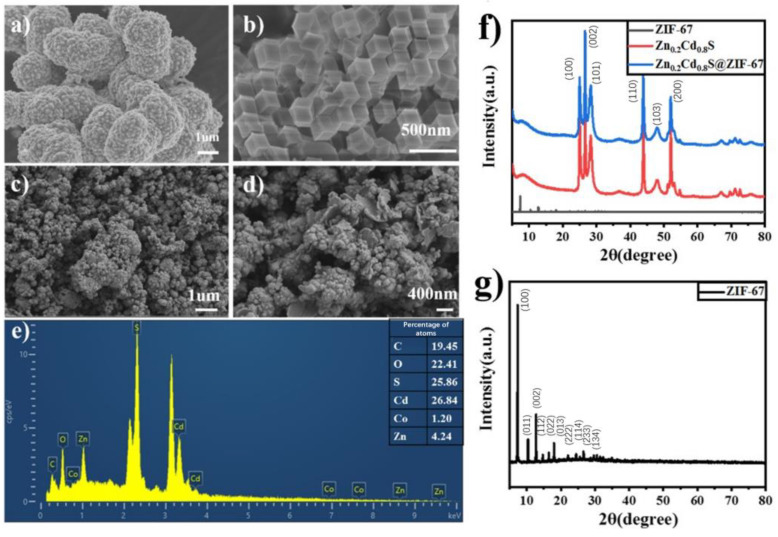
(**a**) SEM image of Zn_0.2_Cd_0.8_S; (**b**) ZIF-67; (**c**,**d**) Zn_0.2_Cd_0.8_S@ZIF-67; (**e**) EDX pattern of Zn_0.2_Cd_0.8_S@ZIF-67; XRD patterns of Zn_0.2_Cd_0.8_S (**f**) and Zn_0.2_Cd_0.8_S@ZIF-67 (**g**).

The SEM images can provide information about the microstructure of ZIF-67, Zn_0.2_Cd_0.8_S, Zn_0.2_Cd_0.8_S@ZIF-67. It can be seen from Figure 1a that Zn_0.2_Cd_0.8_S has flower-like morphology, but with obvious agglomeration. Figure 1b shows the rhombic dodecahedron structure of ZIF-67, which has a smooth surface and large area. In Figure 1c,d, when ZIF-67 is doped in Zn_0.2_Cd_0.8_S, the surface morphology changes. Zn_0.2_Cd_0.8_S@ZIF-67 becomes coarser, and Zn_0.2_Cd_0.8_S increases the surface area. The EDS diagram shows the C, O, S, Cd, Zn, and Co traces (Figure 1e).

The diffraction patterns of Zn_0.2_Cd_0.8_S of are 25.19°, 26.62°, 28.16°, 43.56°, 47.99°, and 51.95° (JCPDS no. 49–1302), corresponding to (100), (002), (101), (110), (103), and (200) planes (Figure 1f). The peaks of Zn_0.2_Cd_0.8_S are sharp, indicating high crystallinity. The diffraction peaks of Zn_0.2_Cd_0.8_S are slightly weaker than those of Zn_0.2_Cd_0.8_S@ZIF-67, indicating a slightly positive effect of ZIF-67 on the Zn_0.2_Cd_0.8_S crystallization. The crystalline size and % crystallinity of Zn_0.2_Cd_0.8_S and Zn_0.2_Cd_0.8_S@ZIF-67 are shown in Table S1. From Figure 1g, the XRD patterns of ZIF-67 show obvious diffraction peaks at 7.46°, 10.47°, 12.52°, 14.76°, 16.50°, 22.40°, 24.56°, and 26.94°, corresponding to (011), (002), (112), (022), (013), (222), (114), (233), and (134) crystal faces, respectively. Since the content of ZIF-67 is small, the corresponding information of the EDS diagram Zn_0.2_Cd_0.8_S@ZIF-67 is weak in Figure 1e. Meanwhile, the diffraction peak intensity of ZIF-67 itself is significantly weaker than that of Zn_0.2_Cd_0.8_S and Zn_0.2_Cd_0.8_S@ZIF-67. Therefore, the peak value of ZIF-67 may also be submerged. The co-catalyst does not reflect the diffraction peak of ZIF-67. 

**Figure 2:**
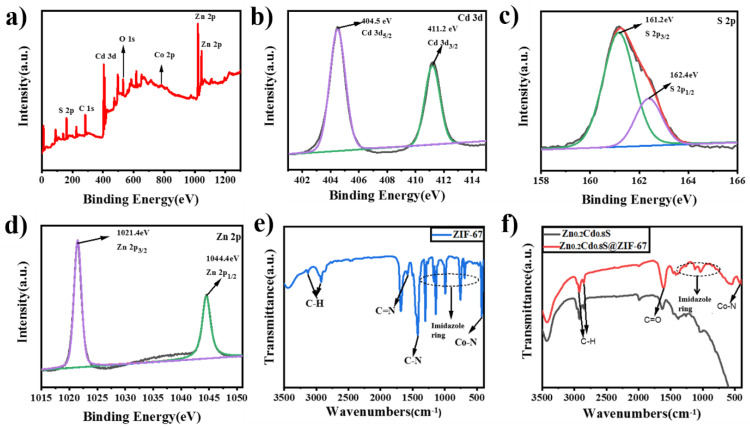
(**a**) Full scan XPS spectrum of Zn_0.2_Cd_0.8_S@ ZIF-67; (**b**) High-resolution XPS Cd 3d; (**c**) Zn 2p; (**d**) S 2p spectrum of Zn_0.2_Cd_0.8_S@ZIF-67; FTIR spectra of (**e**) ZIF-67 and (**f**) Zn_0.2_Cd_0.8_S@ZIF-67.

Element analysis of Zn_0.2_Cd_0.8_S @ZIF-67 was performed using XPS (Figure 2a), in which the distribution of the Co element is very weak compared with the full spectrum of pure Zn_0.2_Cd_0.8_S. The distribution strength of Zn is increased, which may be due to the addition of ZIF-67, increasing the content of Zn in the total proportion. Two peaks of 404.5 eV and 411.2 eV in the XPS spectra of Cd 3d, corresponding to Cd 3d_5/2_, Cd 3d_3/2_ (Figure 2b), were observed in contrast to the fine spectra of Cd 3d XPS in Figure S1. The binding energies of Cd 3d_5/2_ and Cd 3d_3/2_ in the catalyst decreased by 0.1 eV and 0.2 eV, respectively. The peak at 161.2 eV in the S 2p XPS map in Figure 2c belongs to S 2p_3/2_, and the peak at S 2p_1/2_ corresponds to the peak at 162.4 eV. Compared with the fine spectrum of S 2p XPS, the binding energy at S 2p_3/2_ and S 2p_1/2_ increased by 0.1 eV and 0.2 eV, respectively. Figure 2d shows the high-resolution Zn 2p spectra resolved into peaks centered at 1021.4 eV and 1044.4 eV, corresponding to Zn 2p_3/2_ and Zn 2p_1/2_. Compared to the fine spectra of Zn 2p XPS, the peak of Zn_0.2_Cd_0.8_S@ZIF-67 decreased by 0.1 eV. The change of Zn_0.2_Cd_0.8_S@ZIF-67 XPS binding energy may be due to crystalline size and electron concentrations. The electron concentrations are caused by the interface interaction and electron transfer in the heterojunction formed by two semiconductors. It is concluded that ZIF-67 and Zn_0.2_Cd_0.8_S interact with each other. Electron transfer exists between the two photocatalytic semiconductors, which proves that ZIF-67 was successfully complexed with Zn_0.2_Cd_0.8_S. Co 2p can be decomposed into two distinct characteristic peaks: Co 2p_3/2_ corresponds to the binding energy of 781.1 eV, and Co 2p_1/2_ corresponds to the binding energy of 796.2 eV.

As can be seen in Figure 2e, the characteristic peaks at 3137 cm^−1^ and 2925 cm^−1^ correspond to the stretching vibration of C-H of aromatic hydrocarbon and aliphatic hydrocarbon in raw 2-Methylimidazole, respectively. The characteristic peak at 1419 cm^−1^ indicates the stretching vibration of C-N. The stretching vibration of C=N corresponds to the wavenumber 1573 cm^−1^. The wavenumber of 424 cm^−1^ is the characteristic absorption peak belonging to Co-N. The out-of-plane vibration of the imidazole ring is shown by the characteristic peaks at wavenumbers of 1301 cm^−1^, 1139 cm^−1^, 993 cm^−1^, 752 cm^−1^, and 688 cm^−1^. The results above indicate that ZIF-67 was synthesized successfully. The characteristic peaks of 3440 cm^−1^ correspond to the stretching peaks of O-H, while the stretching peaks of 2950 cm^−1^ and 2856 cm^−1^ represent the stretching peaks of C-H (Figure 2f). The wavenumbers of 1623 cm^−1^ and 1378 cm^−1^ are attributed to the stretching vibration of C=O and the stretching vibration of C-OH, respectively. These come from cadmium acetate and zinc acetate. Based on the infrared spectra of Zn_0.2_Cd_0.8_S, the strength of the Zn_0.2_Cd_0.8_S@ZIF-67 at 1606 cm^−1^ C=O tensile vibration peak was obviously increased. The characteristic peak at 1120 cm^−1^ is evident, which corresponds to the plane vibration of the imidazole ring. A characteristic peak appears at about 570 cm^−1^. At the same time, the characteristic peak of Co-N at 418 cm^−1^ is also observed. The above results show that ZIF-67 was successfully compounded on Zn_0.2_Cd_0.8_S.

### 3.2. Photoelectrochemical Performances and Analysis

**Figure 3:**
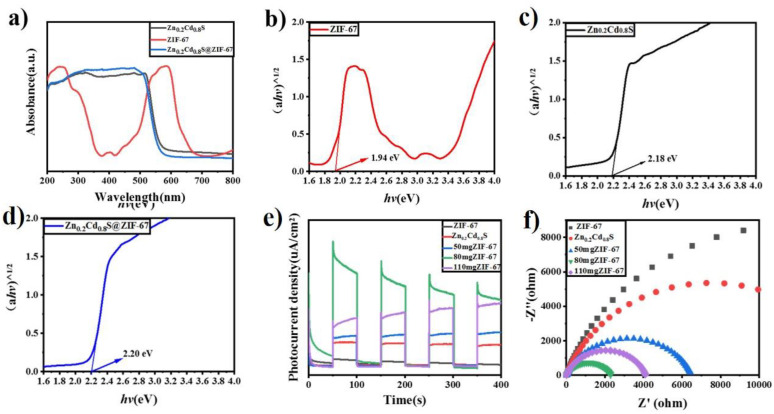
(**a**) UV-Vis DRS absorption spectra of Zn_0.2_Cd_0.8_S, ZIF-67 and Zn_0.2_Cd_0.8_S@ZIF-67; Band gap energy of (**b**) ZIF-67, (**c**) Zn_0.2_Cd_0.8_S and (**d**) Zn_0.2_Cd_0.8_S@ ZIF-67; (**e**) The instantaneous photocurrent diagram of Zn_0.2_Cd_0.8_S @CDs; (**f**) The electrochemical impedance diagram of Zn_0.2_Cd_0.8_S@ ZIF-67.

Both Zn_0.2_Cd_0.8_S and Zn_0.2_Cd_0.8_S@ZIF-67 have absorption in the 200–500 nm wavelength. ZIF-67 has apparent UV absorption at 243 nm and 581 nm, as shown in Figure 3a. The bandgap of ZIF-67 is 1.94 eV (Figure 3b), and the bandgap of Zn_0.2_Cd_0.8_S is 2.18 eV (Figure 3c). The bandgap of Zn_0.2_Cd_0.8_S@ZIF-67 is 2.20 eV (Figure 3d), only increased to 0.2 eV. It can be seen that ZIF-67 can improve the light absorption capacity of Zn_0.2_Cd_0.8_S, but has little effect on the band gap. This is due to strong interaction and coupling effects of Zn_0.2_Cd_0.8_S and ZIF-67 at the interface. These effects decrease the recombination probability of electrons and holes.

The photocatalytic activity of Zn_0.2_Cd_0.8_S@ZIF-67 was analyzed by an electrochemical experiment. In Figure 3e, ZIF-67, Zn_0.2_Cd_0.8_S, and Zn_0.2_Cd_0.8_S@ZIF-67 generate photocurrents before and after the light source is turned on. The photocurrent signal of Zn_0.2_Cd_0.8_S@ZIF-67 was more substantial than that of a single catalyst, and ZIF-67 was the weakest. When the additional amount of ZIF-67 is 80 mg, the fast photocurrent reaches the maximum. This indicates that the photocatalysis performance is the best. ZIF-67 has a large surface area, providing more photocatalytic active sites for Zn_0.2_Cd_0.8_S. It can better disperse Zn_0.2_Cd_0.8_S and inhibit Zn_0.2_Cd_0.8_S agglomeration. Thus, the light absorption ability of Zn_0.2_Cd_0.8_S is improved. The existence of ZIF-67 is not only beneficial to the absorption of light by the catalyst, but also reduces the recombination of photoexcited electrons and holes. These effects shorten the transfer distance of charge carriers between the valence band and conduction band. The coordinated bandgap properties and close interaction between ZIF-67 and Zn_0.2_Cd_0.8_S provide a new path for charge transfer.

The electrochemical impedance measurement is consistent with the instantaneous photocurrent measurement, as shown in Figure 3f. When the addition of ZIF-67 was 80 mg, the Nyquist diagram had the smallest arc radius, the slightest resistance, and the best photocatalytic performance. The results showed that ZIF-67 doped with Zn_0.2_Cd_0.8_S could expose more catalytic active sites and promote the absorption of light energy.

The electron transfer behavior of ZIF-67, Zn_0.2_Cd_0.8_S, and Zn_0.2_Cd_0.8_S@ZIF-67 can be seen in Figure S2. Their separation abilities of light-generating carriers were characterized by fluorescence spectroscopy (PL). A high-intensity PL characteristic peak appears at 422 nm. The high electron-hole recombination ratio of ZIF-67 results in the intense luminescence emission of ZIF-67. Compared with ZIF-67, the fluorescence intensity of Zn_0.2_Cd_0.8_S and Zn_0.2_Cd_0.8_S@ZIF-67 decreased significantly. This indicates that the carrier separation efficiency of these photocatalysts was higher than that of ZIF-67. Because of the doping of ZIF-67 into Zn_0.2_Cd_0.8_S, the crystalline size changed. The number of trapping sites of the luminescent electrons in Zn_0.2_Cd_0.8_S@ZIF-67 is reduced. This results in a lower luminous peak value for Zn_0.2_Cd_0.8_S@ZIF-67. Band gap characteristics and crystalline size changes of Zn_0.2_Cd_0.8_S@ZIF-67 increase the separating photogenerated electron and electron-hole pairs. This activity will further facilitate the photocatalyst degradation.

### 3.3. Photocatalyst Degradation of RhB

**Figure 4:**
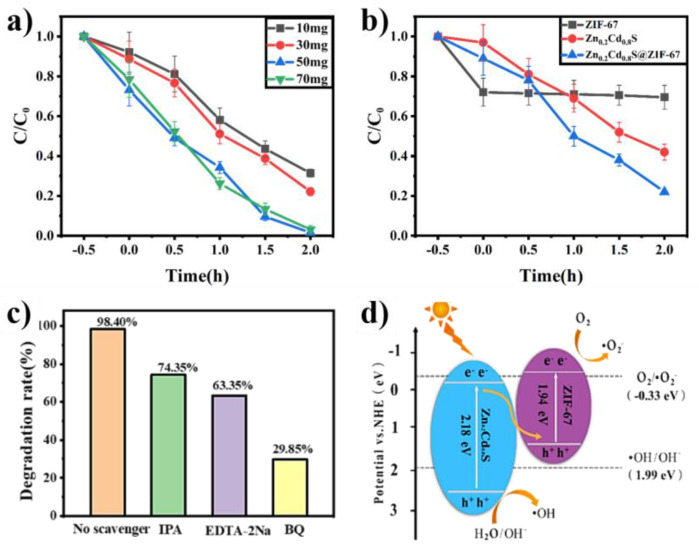
(**a**) C/C_0_ of RhB varied with Zn_0.2_Cd_0.8_S@ZIF-67; (**b**) C/C_0_ of RhB with ZIF-67, Zn_0.2_Cd_0.8_S, and Zn_0.2_Cd_0.8_S@ZIF-67; (**c**) Experiment of RhB capture by Zn_0.2_Cd_0.8_S@ZIF-67 under Ultraviolet radiation. (**d**) Proposed Z-type photocatalytic system of Zn_0.2_Cd_0.8_S@ZIF-67 under ultraviolet radiation.

The photocatalytic degradation of RhB on Zn_0.2_Cd_0.8_S@ZIF-67 was carried out at 10, 30, 50, and 70 mg, respectively (Figures S3 and S4). The C/C_0_ of RhB with Zn_0.2_Cd_0.8_S@ZIF-67 decreased with the illumination time (Figure 4a,b). In the dark adsorption equilibrium experiment, we can see that the degradation of RB increases from 10 to 50 mg. When the content of ZIF-67 was increased to 70 mg, C/C_0_ decreased slightly. The best degradation performance was achieved under the addition of 50 and 70 mg of catalyst. C/C_0_ was 98.40%. The large specific surface area of ZIF-67 can not only provide more active centers for the adsorption of reactants on the surface of the heterogeneous photocatalyst, but also shorten the transmission distance of carriers. A sufficient number of Zn_0.2_Cd_0.8_S@ZIF-67 can reduce the occurrence of electron-hole pair recombination and accelerate the movement of charges between catalysts. 

The C/C_0_ of ZIF-67, Zn_0.2_Cd_0.8_S, and Zn_0.2_Cd_0.8_S@ZIF-67 are shown in Figure 4b. The mass of three catalysts were 30 mg. The concentration of RhB was selected to be 10 mg/L. In the dark environment, the C/C_0_ of RhB by ZIF-67 is the highest, reaching 28.00%. The second is Zn_0.2_Cd_0.8_S@ZIF-67, with C/C_0_ of RhB increased to 11.35%. The lowest is C/C_0_ of RhB by Zn_0.2_Cd_0.8_S, which is only 5.70%. Under visible light irradiation, ZIF-67 has little fluctuation in C/C_0_ of RhB, about 30%. This indicates that light has little effect on its behavior. The degradation performance of RhB by Zn_0.2_Cd_0.8_S and Zn_0.2_Cd_0.8_S@ZIF-67 showed an upward trend with the increase of light time. The degradation performance was the largest when the illumination time was between 0.5 h and 1 h. During this time, C/C_0_ reached 48.85% from 23.35%. Within 2 h, it reached 77.85%. On the one hand, this may be due to the morphological change of Zn_0.2_Cd_0.8_S@ZIF-67 (as shown in SEM images in Figure 1c). The addition of ZIF-67 to Zn_0.2_Cd_0.8_S results in smaller sized nanomaterials. On the other hand, the slightly larger crystal size of Zn_0.2_Cd_0.8_S (shown in XRD results in Figure 1f) facilitated the electron transfer. The large surface area and mesoporous structure strengthens its adsorption and degradation ability. The electron movement space is wider, and the electron-hole pair becomes difficult to recombine, thereby improving the degradation performance of RhB. 

As can be seen from Figure 4c, the final C/C_0_ of RhB was 98.40% in RhB solution without a trapping agent, 74.35% in RhB solution with the trapping Agent IPA, and 63.35% in RhB solution with EDTA-2Na. The addition of the capture agent BQ resulted in the worst degradation of RhB (29.85%), which was significantly less than that of no capture agent. The photocatalysis mechanism of Zn_0.2_Cd_0.8_S@ZIF-67 in photocatalytic degradation of RHB was further investigated (Figure 4d). EDTA-2Na is used as an h+ trapping agent, BQ as ·O_2_^−^ trapping agent, and IPA as ·OH trapping agent. It can be found that ·O_2_^−^ plays a significant role in the photocatalytic degradation of RhB. The following is h+, and finally, ·OH scavenger.

The conduction band (CB) and valence band (VB) potentials of Zn_0.2_Cd_0.8_S and ZIF-67 were estimated by electrochemical workstation, and Mott–Schottky diagrams were obtained (Figure S4). Both Zn_0.2_Cd_0.8_S and ZIF-67 are n-type semiconductors [21,22]. The flat band potential of Zn_0.2_Cd_0.8_S is estimated to be −0.27 eV [23], and ZIF-67 is −0.72 eV [24] by the Mott–Schottky method. For n-type semiconductors, the flat band potential is generally 0.1 eV more positive than the conduction potential [25]. Compared with the standard hydrogen electrode (0.24 eV, NHE), the conduction potential of Zn_0.2_Cd_0.8_S is calculated to be E_CB_ = −0.13 eV, and the conduction potential of ZIF-67 is −0.58 eV (Figure S4). The valence band (E_VB_) is derived from the formula E_VB_ = E_G_ + E_CB_, the valence band of Zn_0.2_Cd_0.8_S is 2.05 eV [26,27], and the valence band of ZIF-67 is 1.36 eV.

According to the derivation, the ZIF-67 valence band potential (1.36 eV) is lower than the standard OH potential (1.99 eV) [28], and the hole from the ZIF-67 valence band cannot oxidize the water molecule to ·OH. This suggests that the holes in the ZIF-67 valence band do not have enough energy to oxidize OH- or H_2_O to ·OH. The electrons in the Zn_0.2_Cd_0.8_S conduction band do not have enough energy to reduce O_2_ to ·O_2_^−^ because the potential of the Zn_0.2_Cd_0.8_S conduction band is higher than E_θ_ (O_2_/·O_2_^−^) (−0.33 eV, NHE). The h^+^, ·O_2_^−^, and ·OH are the main active substances in the degradation of RhB [29,30]. The type II heterostructure system [31] obviously cannot explain the photoelectron and hole transfer process of Zn_0.2_Cd_0.8_S@ZIF-67 [32,33].

Based on the above derivation results, we propose a Z-type photocatalytic mechanism. Under visible light irradiation, the Zn_0.2_Cd_0.8_S and ZIF-67 conduction bands are excited to generate electrons, and holes are generated on the valence band. CB (−0.58eV) of ZIF-67 is lower than E_θ_ (O_2_/·O_2_^−^) (−0.33eV). Therefore, photogenerated electrons reduce O_2_ to form the active substance (·O_2_^−^). The VB (2.05 eV) potential of Zn_0.2_Cd_0.8_S is higher than that of E_θ_ (·OH/H_2_O) (1.99 eV), which means that photogenerated holes will generate active substances and (·OH) from OH^−^. At the same time, some electrons in the CB of Zn_0.2_Cd_0.8_S will quickly bind to the holes in the VB of ZIF-67. This means that the electrons in the ZIF-67 conduction band have enough energy to reduce O_2_ to O_2_^−^. The holes in the Zn_0.2_Cd_0.8_S valence band have enough energy to oxidize H_2_O to OH. Ultimately, ·OH and ·O_2_^−^ as active species may be involved in photooxidation and degradation of RhB. The heterojunction between Zn_0.2_Cd_0.8_S and ZIF-67 can significantly enhance the separation of e^−^/h^+^ and improve charge transfer at the interface. The formation of this heterojunction can not only accelerate the separation and transfer rate of photogenerated charges, but also maintain a strong redox ability and effectively degrade the dye RhB.

### 3.4. DA Sensor and Mechanism Exploration

**Figure 5:**
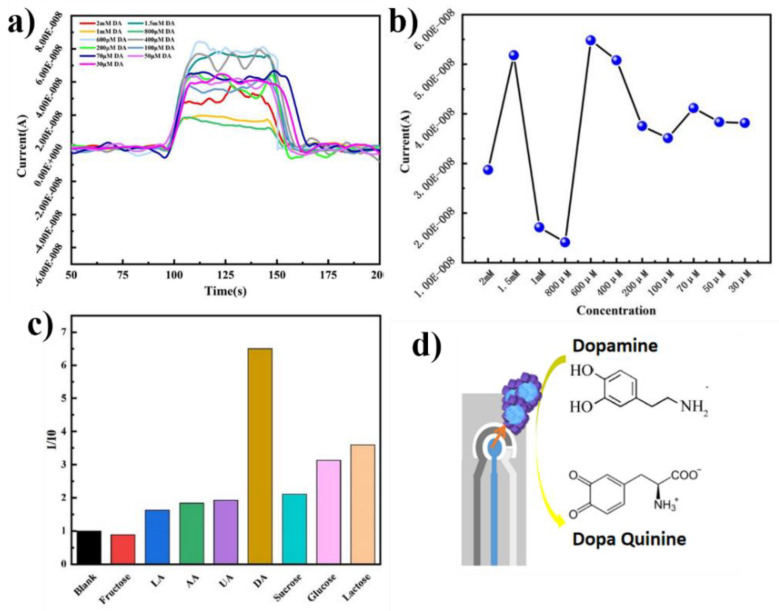
(**a**) DA sensing photocurrent response; (**b**) the sensitivity and (**c**) selectivity of the Zn_0.2_Cd_0.8_S@ZIF-67 to DA; (**d**) photocatalytic mechanism of Zn_0.2_Cd_0.8_S@ZIF-67 as DA sensor.

Zn_0.2_Cd_0.8_S@ZIF-67 was dissolved in a mixture of ethanol and Nafion and sonicated. Suspension droplets were applied to a clean screen-printed electrode (SPCE) and dried. Zn_0.2_Cd_0.8_S@ZIF-67/SPCE was formed and used to measure the photocurrent of DA hydrochloride under illumination. In Figure 5a, the current changes at different DA concentrations under light irradiation are presented. At the constant DA concentration, with the light on at 100 s, the current increased to a plateau and maintained constance. When the light turned off after 50 s, the detection current decreased to the previous base line. In Figure 5b, the DA with the concentration of 30 M to 2 mM can be detected. It was found that polydopamine produced by oxidation of DA could effectively enhance the photocurrent. However, it is notable that the detection current is not high enough for a concentration-dependent analysis. This may be due to the morphology of Zn_0.2_Cd_0.8_S@ZIF-67. The 0D Zn_0.2_Cd_0.8_S particles combined with 3D ZIF-67 shells inhibit the charge transfer within the system. Additional 1D and 2D nanomaterials might be a solution for bridging the charge transfer between 0D and 3D nanomaterials. 

The selectivity of Zn_0.2_Cd_0.8_S@ZIF-67 toward DA was evaluated by using a CHI760E platform. In human blood plasma, DA co-exists with many kinds of biomolecules, such as AA, UA, and glucose. It is essential to confirm the ability of the catalyst for the selection detection of DA. Herein, we selected fructose, LA, AA, UA, sucrose, glucose, and lactose as the interfering molecules toward DA. As shown in Figure 5c, the amperometric current change is evident with the addition of DA, while there is relatively little change with the addition of the same amount interfering molecules. Taken together, we can come to the conclusion that Zn_0.2_Cd_0.8_S@ZIF-67 is a promising platform for DA detection. 

Although the detection sensitivity is not high enough, Zn_0.2_Cd_0.8_S@ZIF-67 displayed outstanding selectivity toward DA. Photocurrent promotion toward DA may be attributed to the interaction between photogenerated electrons and electron receptors (Figure 5d). On the one hand, through radical capture experiments, superoxide radicals (·O_2_^−^) played a major role in the DA sensing. On the other hand, the structure of ZIF-67 can provide more photocatalytic activity check points for Zn_0.2_Cd_0.8_S and enhance the sensing efficiency of DA. In addition, the heterojunction between Zn_0.2_Cd_0.8_S and ZIF-67 can significantly enhance the separation of e^−^/h^+^ and improve charge transfer at the PEC interface. Therefore, in the presence of DA hydrochloride, the two free phenolic hydroxyl groups can act as an effective electron donor for the photoexcited Zn_0.2_Cd_0.8_S@ZIF-67 and promote charge transfer from Zn_0.2_Cd_0.8_S to ZIF-67, thus resulting in the anodic photocurrent and DA sensing. The results provide a possibility to construct the PEC sensor platform for DA detection.

## 4. Conclusions

In this study, Zn_0.2_Cd_0.8_S@ZIF-67 was prepared via a hydrothermal method. The photocurrent intensity of Zn_0.2_Cd_0.8_S@ZIF-67 was the highest, and the electrode resistance was the lowest, when the addition of ZIF-67 reached 80 mg. Meanwhile, Zn_0.2_Cd_0.8_S@ZIF-67 shows lower fluorescence intensity in PL spectra, indicating fewer electron-hole recombination. When the dosage of Zn_0.2_Cd_0.8_S@ZIF-67 reached 50 mg, the degradation percentage reached the maximum of 98.40%. In addition, a superoxide radical (·O_2_^−^) plays a major role in the degradation of RhB, confirmed by free radical capture experiments. Finally, the DA sensor modified with Zn_0.2_Cd_0.8_S@ZIF-67 exhibits excellent selectivity and high response within the DA concentration of 30 M to 2 mM. This study provides strategies for developing cost-efficient, highly selective DA detection methods.

## Supplementary Materials

The following supporting information can be downloaded at: https://www.mdpi.com/article/10.3390/ma15217683/s1, Figure S1: High-resolution XPS spectrum; Figure S2: Steady-state fluorescence spectra; Figure S3: UV-Vis spectrogram; Figure S4: The pseudo-first-order kinetics of RhB; Figure S5: Mott–Schottky plots; Table S1: XRD peaks analysis.
