# Supplementary material for: Heterostructured ZnCdS@ZIF-67 as a Photocatalyst for Fluorescent Dye Degradation and Selectively Nonenzymatic Sensing of Dopamine
Source: Materials (Basel). 2022 Nov 1;15(21):7683. doi: 10.3390/ma15217683 (PMC9655990; doi:10.3390/ma15217683)
Supplement: Supplementary file 1 [file materials-15-07683-s001.zip › materials-1958101-supplementary.pdf]

Supporting Information

Heterostructured ZnCdS@ZIF-67 as photocatalyst for fluorescent dye degradation and selectively nonenzymatic sensing of dopamine

Zhichao Wang[1], Bianying Wen[,2], Jie Zhou[3], Xin Zhao[3], Xiaoyuan Zhang*[3], Zhiqiang Su*[3]*

1. Precision Forestry Key Laboratory of Beijing, Beijing Forestry University, Beijing 100083, China.
2. Key Laboratory of Processing and Quality Evaluation Technology of Green Plastics of China National Light Industry Council, Beijing Technology and Business University, Beijing 100048, China
3. State Key Laboratory of Chemical Resource Engineering, Beijing Key Laboratory of Advanced Functional Polymer Composites, Beijing University of Chemical Technology, Beijing 100029, China.

* Corresponding authors. E-mail addresses: weibianying@tsinghua.org.cn (B. Wen), 2020700036@buct.edu.cn (X. Zhang), suzq@mail.buct.edu.cn (Z. Su)

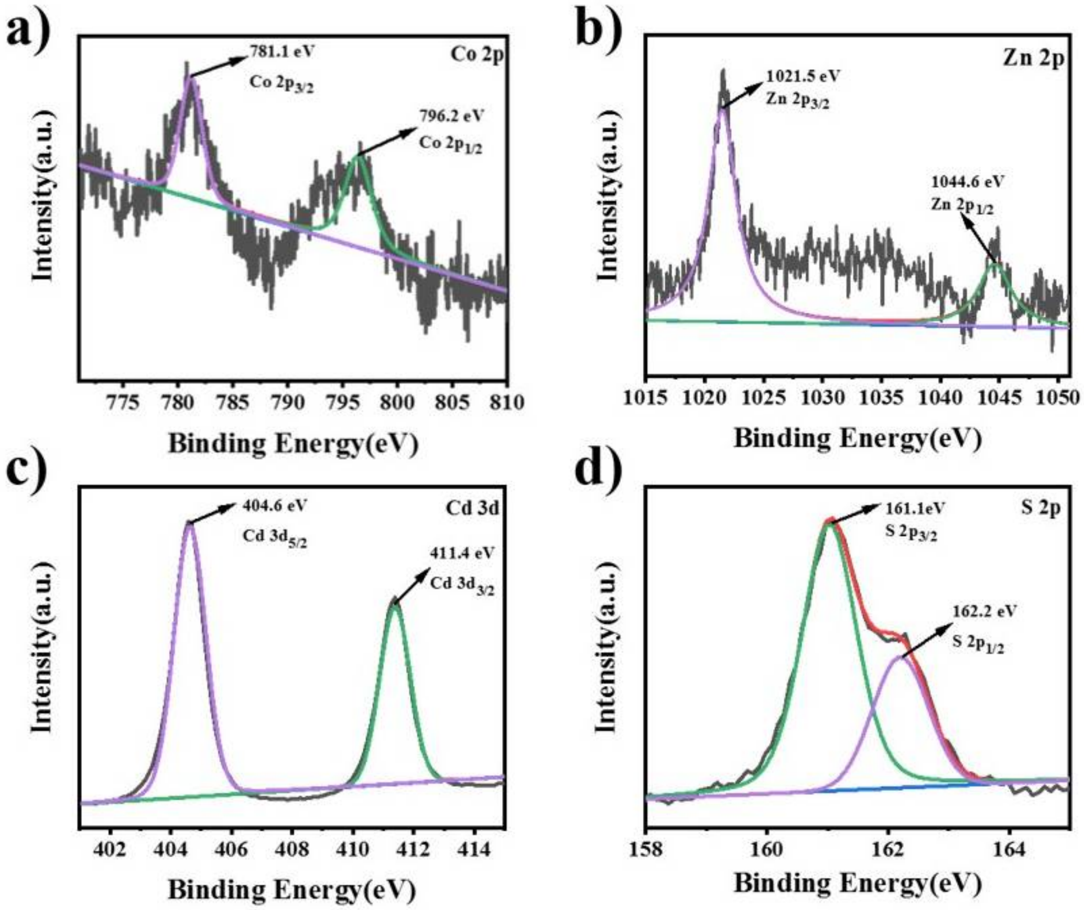

Figure S1. High-resolution XPS Co 2p (**a**)spectrum of $Zn_{0.2}Cd_{0.8}S$@ ZIF-67; Zn 2p (**b**); Cd 3d (**c**); S 2p (**d**)spectrum of $Zn_{0.2}Cd_{0.8}S$.

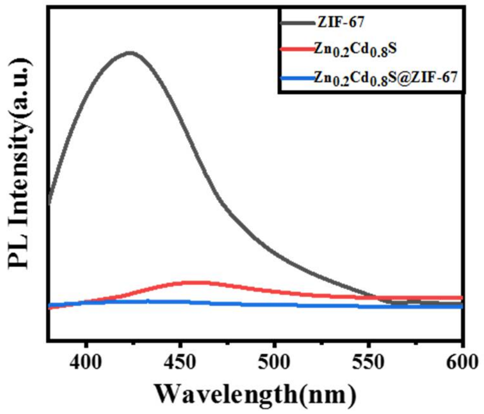

Figure S2. Steady-state fluorescence spectra of $Zn_{0.2}Cd_{0.8}S$, ZIF-67 and $Zn_{0.2}Cd_{0.8}S$@ ZIF-67.

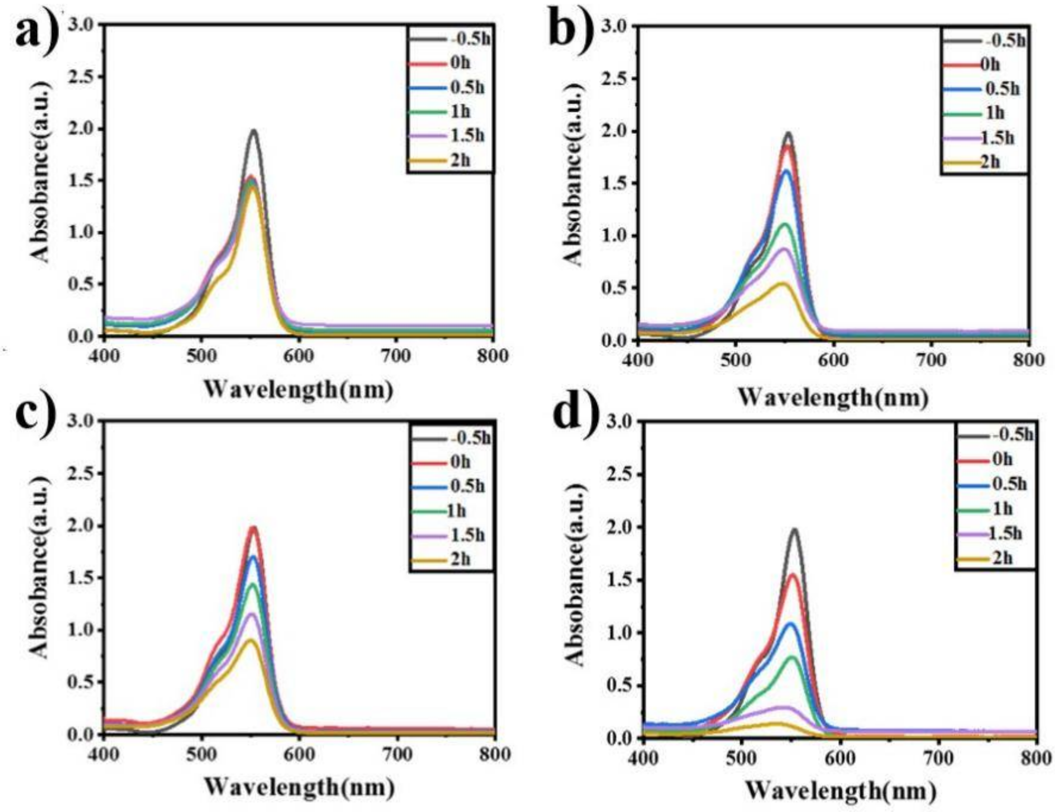

Figure S3. UV-Vis spectrogram of RhB degradation by (**a**)$Zn_{0.2}Cd_{0.8}S$, (**b**)ZIF-67, (**c**)$Zn_{0.2}Cd_{0.8}S$@ZIF-67 and (**d**) $Zn_{0.2}Cd_{0.8}S$@ZIF-67(50 mg).

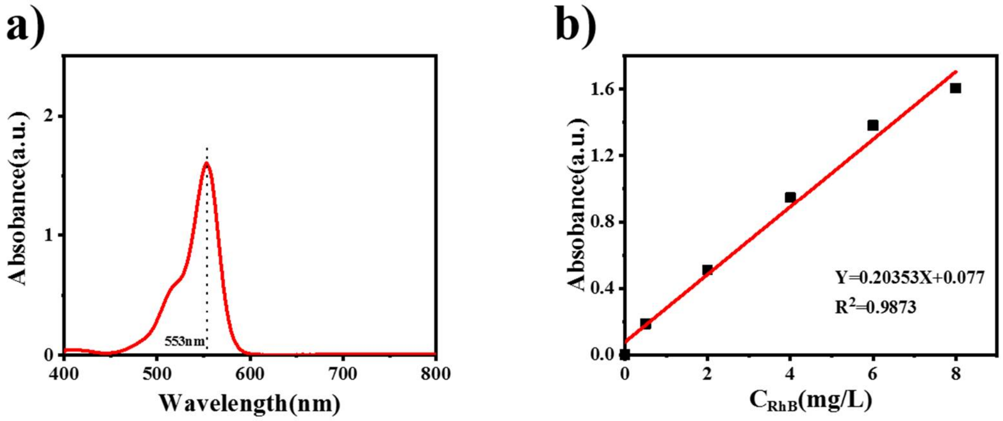

Figure S4. (**a**) The absorbance of RhB; (**b**) The pseudo-first-order kinetics of RhB used for $Zn_{0.2}Cd_{0.8}S$@ZIF-67.

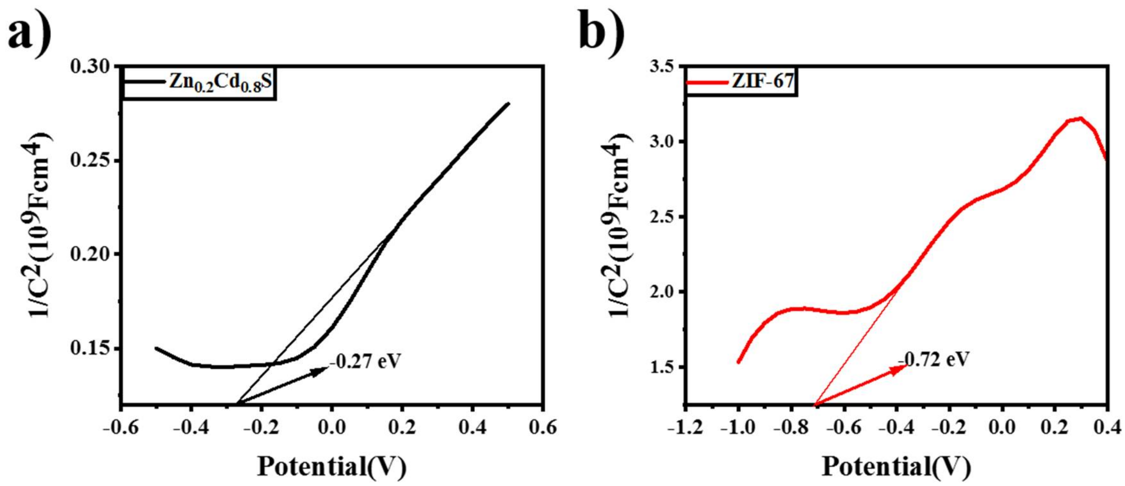

Figure S5. Mott-schottky plots of (**a**)$Zn_{0.2}Cd_{0.8}S$ and (**b**) ZIF-67.

Table S1. XRD peaks analysis for $Zn_{0.2}Cd_{0.8}S$ and $Zn_{0.2}Cd_{0.8}S$@ZIF-67.

Sample	Peak/°	hkl	crystalline size (A)	% crystallinity
**$Zn_{0.2}Cd_{0.8}S$**	24.915	100	3.5708	69.8
	28.124	101	3.1703	100
	51.659	200	1.7679	95.2
**$Zn_{0.2}Cd_{0.8}S$@ZIF-67**	24.847	100	3.5804	78.7
	26.595	002	3.3489	70.6
	28.258	101	3.1555	96.4
	43.669	110	2.0711	93.5
	48.140	103	1.8886	5.8
	51.905	200	1.7601	7.2
